# Oxidized Low-Density Lipoprotein Suppresses Expression of Prostaglandin E Receptor Subtype EP3 in Human THP-1 Macrophages

**DOI:** 10.1371/journal.pone.0110828

**Published:** 2014-10-21

**Authors:** Xuxia Sui, Yanmin Liu, Qi Li, Gefei Liu, Xuhong Song, Zhongjing Su, Xiaolan Chang, Yingbi Zhou, Bin Liang, Dongyang Huang

**Affiliations:** 1 Department of Cell Biology, Key Laboratory of Molecular Biology in High Cancer Incidence Coastal Chaoshan Area of Guangdong Higher Education Institutes, Shantou University Medical College, Shantou, Guangdong, China; 2 Department of Cardiovascular Research Center, Shantou University Medical College, Shantou, Guangdong, China; Nihon University School of Medicine, Japan

## Abstract

EP3, one of four prostaglandin E2 (PGE2) receptors, is significantly lower in atherosclerotic plaques than in normal arteries and is localized predominantly in macrophages of the plaque shoulder region. However, mechanisms behind this EP3 expression pattern are still unknown. We investigated the underlying mechanism of EP3 expression in phorbol 12-myristate 13-acetate (PMA)-differentiated THP-1 macrophages with oxidized low-density lipoprotein (oxLDL) treatment. We found that oxLDL decreased EP3 expression, in a dose-dependent manner, at both the mRNA and protein levels. Moreover, oxLDL inhibited nuclear factor-κB (NF-κB)-dependent transcription of the EP3 gene by the activation of peroxisome proliferator-activated receptor-γ (PPAR-γ). Finally, chromatin immunoprecipitation revealed decreased binding of NF-κB to the EP3 promoter with oxLDL and PPAR-γ agonist treatment. Our results show that oxLDL suppresses EP3 expression by activation of PPAR-γ and subsequent inhibition of NF-κB in macrophages. These results suggest that down-regulation of EP3 expression by oxLDL is associated with impairment of EP3-mediated anti-inflammatory effects, and that EP3 receptor activity may exert a beneficial effect on atherosclerosis.

## Introduction

Atherosclerosis is the leading cause of death in industrialized societies, and is a non-resolving inflammatory disease [1], [2]. The early stages of this disorder involve formation of cholesterol-rich lesions beneath the arterial endothelium, leading to the migration of circulating monocytes into the vessel wall and their subsequent differentiation into macrophages. Macrophages ingest large amounts of lipids and modified lipoproteins, e.g. oxLDL, in an uncontrolled manner, leading to the formation of foam cells, the major cellular component of fatty streaks [3].

Macrophages play a central role in the development of atherosclerosis by producing a variety of mediators, including prostaglandin E2 (PGE2) [4]. PGE2 is a dual-function prostanoid and has been reported to have both pro- and anti-inflammatory effects [5], and mediates its various actions via binding to 4 receptors (EP1, EP2, EP3 and EP4) [6]. EP1 and EP3 inhibit adenylate cyclase and decrease cAMP levels, whereas EP2 and EP4 stimulate adenylate cyclase and increase cAMP levels [7], [8]. It is known that activation of EP2 and EP4 exerts pro-inflammatory effects in atherosclerotic plaques [9], [10]. However, the role of the EP3 receptor in atherosclerotic plaques has received much less attention. EP3 expression is significantly lower in atherosclerotic plaques than in normal arteries, and is localized mainly in macrophages of the plaque shoulder region [11], [12], [13]. However, mechanisms that regulate EP3 expression are still unclear.

OxLDL regulates macrophage gene expression through ligand activation of PPAR-γ, which plays a key role in adipocyte differentiation and lipid storage by regulating the expression of genes critical for adipogenesis [14], [15]. 15-deoxy-D^12,14^-prostaglandin J_2_ (15d-PGJ2) and troglitazone, respectively, are the most widely used natural and synthetic agonists for PPAR-γ [16], [17], whereas GW9662 and T0070907 (T007) are potent, selective antagonists of the PPAR-γ receptor [18], [19]. Activation of PPAR-γ inhibits the expression of various macrophage cytokines by antagonizing the transcription factor NF-κB [20], [21]. In vertebrates, this family comprises p65, p50, p52, c-Rel and RelB. 3 of the members (p65, RelB and c-Rel) have a transactivation domain in their C terminus that forms various homodimers and heterodimers with the other two proteins; the most common and most widely studied form is the p65 subunit of the p50/p65 heterodimer [22]. NF-κB is present in the cytoplasm in an inactive state bound to an inhibitory protein known as IκB. Treatment of cells with various inducers results in the degradation of IκB proteins and the bound NF-κB is released and translocates to the nucleus to activate target genes [23]. NF-κB can activate multiple inflammatory genes and plays an important role in atherosclerosis [24]. NF-κB and EP3 proteins co-localize in plaque cells and NF-κB inhibitors reduce EP3 expression in THP-1 cells [11].

Human THP-1 monocytic leukemia cells were induced to differentiate into macrophages by PMA and then treated with oxLDL to form foam cells, as previously described [25], [26]. In the present study, we investigated the regulatory mechanism by which oxLDL suppresses EP3 expression and characterized the effects of NF-κB and PPAR-γ on EP3 expression in PMA-differentiated macrophages.

## Materials and Methods

### Materials

Human THP-1 monocytic leukemia cells were from the Shanghai Cell Institute, Chinese Academy of Science. RPMI 1640 medium, fetal bovine serum (FBS) and penicillin and streptomycin solution were from Hyclone (Logan, UT, USA). OxLDL was from Yiyuan Biotechnologies (Guangzhou, China). PMA, 3,3′-diaminobenzidine tetrahydrochloride (DAB), 15d-PGJ2, troglitazone, parthenolide, GW9662, T007 and β-actin antibody were from Sigma-Aldrich (St. Louis, MO, USA). EP3 antibody was from Santa Cruz Biotechnology (Santa Cruz, CA). NF-κB p65 antibody was from Abcam (Cambridge, MA, USA). CD68 antibody was from ZSGB-BIO (Guangzhou, China).

### Cell culture and induction of differentiation

THP-1 cells were cultured in RPMI 1640 medium containing 10% FBS supplemented with penicillin (100 U/ml) and streptomycin (100 µg/ml) at 37°C in 5% CO_2_. THP-1 monocytes were induced to differentiate into macrophages, according to a previously reported protocol [27], [28]. THP-1 monocytes were treated with 0.2 µM PMA for 24 h to allow differentiation into macrophages. The differentiated macrophages were washed 3 times with phosphate-buffered saline (PBS) and incubated with cell culture medium for 24 h at 37°C until the addition of oxLDL (25–100 µg/ml), troglitazone (10 µM), 15d-PGJ2 (1 µM), parthenolide (5 µM), GW9662 (5 µM) or T007 (5 µM) in fresh serum-containing medium. THP-1 macrophages were pretreated with PPAR-γ antagonists T007 (5 µM) or GW9662 (5 µM) for 3 h and then exposed to oxLDL for 24 h. THP-1 macrophages treated with and without oxLDL were stained with Oil-red O to observe intracytoplasmic lipid droplets. To address the complexity of different signals occurring *in vivo* and to maintain a macrophage-like phenotype of differentiated THP-1 cells, all of our studies were performed with serum. PMA was solubilized in dimethyl sulfoxide (DMSO) as a 4 mM stock solution. Troglitazone, GW9662, T007, and parthenolide were solubilized in DMSO as stock solutions of 20 mM, 50 mM, 10 mg/ml, and 100 mM, respectively. Cell viability, determined by MTT, was not decreased by any of the treatments.

### Real-time PCR

Total RNA was isolated from differentiated THP-1 cells with TRIzol reagent (Invitrogen, Carlsbad, CA). Total RNA was reverse-transcribed into single-stranded cDNA using an RT reagent kit (TaKaRa, Shiga, Japan). Real-time PCR involved the use of ABI Prism 7000 (Applied Biosystems) and SYBR Premix Ex Taq (TaKaRa, Shiga, Japan). The amplification program was 95°C for 30 s and 40 cycles at 95°C for 5 s and 60°C for 31 s. The primer sequences for the EP3 receptor and β-actin are listed in Table 1. Primers crossed an exon-exon boundary to ensure that genomic DNA was not amplified. To assess the specificity of each primer set, amplicons generated from the PCR reaction were analyzed by melting point curves and run on 2% agarose gels to confirm the correct sizes of PCR products.

**Table 1:** Primers for the human EP3 receptor and β-actin.

Primer	Sequence (5′-3′)
EP3 Fwd	GTGCTGTCGGTCTGCTG^a^
EP3 Rev	GCCAGGCGAACAGCTATTAAGAAG
β-actin Fwd	ATTGCCGACAGGATGCAGAA^b^
β-actin Rev	GCTGATCCACATCTGCTGGAA^b^

a and b, primers are from references [30] and [31], respectively.

### Western blot analysis

Treated cells were washed 3 times with PBS and lysed in RIPA lysis buffer (25 mM Tris, pH 7.6, 150 mM NaCl, 1% Nonidet P-40, 1% sodium deoxycholate and 0.1% sodium dodecyl sulfate) with protease cocktail inhibitors on ice for 30 min. The resulting cell lysates were clarified by centrifugation at 12,000 g for 15 min at 4°C. The supernatant underwent 10% SDS-PAGE and was then transferred to polyvinylidene difluoride membranes (Millipore, Billerica, MA) at 100 mA for 110 min on ice. The membranes were blocked with 5% non-fat milk in Tris-buffered saline with Tween 20 (TBST; 100 mM Tris-HCl, pH 7.5, 150 mM NaCl and 0.1% Tween 20) and then incubated with a rabbit polyclonal antibody for EP3 (1∶700 in TBST) or a mouse monoclonal antibody for β-actin (1∶2,500 in 5% nonfat milk in TBST) overnight at 4°C. After 3 washes in TBST, membranes were exposed to horseradish peroxidase-conjugated secondary antibody (1∶2,500; Sigma) for 1 h at room temperature. Proteins were detected by enhanced chemiluminescence (Pierce, Rockford, USA) and were visualized using a Genegnom chemiluminescence detection system (Synoptics, Ltd., UK).

### Immunohistochemistry and immunocytochemistry

For immunohistochemistry, paraffin-embedded samples from human atherosclerotic plaques of arch aortas, collected from autopsies, were kindly provided by Prof. Dongping Tian in the Department of Pathology of Shantou University Medical College. This study was approved by the Ethics Committee of Shantou University Medical College. Informed consent was given by their guardians. This study protocol conformed to the ethical guidelines of the 1975 Declaration of Helsinki as reflected in a prior approval by the Ethics Committee of Shantou University Medical College. The arch aorta was sectioned into 4-µm-thick pieces, dewaxed, and rehydrated. After incubation with 2% BSA for 30 min, sections were incubated with primary antibody against NF-κB p65 and EP3 (1∶50 diluted in PBS) overnight at 4°C. After washing with PBS 3 times, cells were incubated with peroxidase-labeled secondary antibodies (1∶500) for 1 h at room temperature. Antibody binding was visualized by DAB (0.5 mg/ml). Slides were then lightly counterstained with hematoxylin and photographed by light microscopy (Olympus BX51). For negative controls, PBS was used instead of the primary antibody.

For immunocytochemistry, THP-1 cells differentiated on chamber slides were treated with the appropriate reagent. After fixing the cells in 4% paraformaldehyde for 10 min, cells were permeabilized in 0.2% Triton X-100 for 10 min and incubated with 2% BSA for 30 min. Cells were then incubated with primary antibody against NF-κB (1∶50 diluted in PBS) or CD68 (1∶100 diluted in PBS) overnight at 4°C. The remainder of the procedure was the same as for immunohistochemistry. The percentage of positive cells per slide was evaluated by counting the number of positive cells and the total number of cells in a minimum of three fields (over 100 cells per slide) under a 20X objective.

### Chromatin immunoprecipitation (ChIP)

ChIP involved the use of the EZ-Magna ChIP Kit (Millipore, Billerica, MA). Briefly, harvested cells were fixed with formaldehyde (final concentration 1%) and the unreacted formaldehyde quenched in glycine. Cells were then suspended in nuclear lysis buffer and sonicated until DNA was fragmented into lengths of between 200 and 1,000 bp. Aliquots (1%) of sheared DNA were removed as input, and the remainder was immunoprecipitated with the rabbit polyclonal antibody for the NF-κB p65 subunit or normal rabbit IgG as a negative control overnight at 4°C with rotation. The samples and input were eluted for reverse crosslinking of protein/DNA. After DNA purification, PCR was used to detect the EP3 promoter and real time-PCR was used to analyze changes in NF-κB binding to the EP3 promoter.

PCR involved the use of a thermal cycler with a 20-µL reaction volume containing 2 µL cDNA, 2.5 units Taq DNA polymerase, and 0.5 mM each of sense and antisense primers. Amplification was 30 cycles (95°C for 30 s, 65°C for 30 s and 72°C for 30 s). PCR products were analyzed by electrophoresis on a 2% agarose gel and visualized with ethidium bromide.

Primers specific for the EP3 promoter were designed based on a previous study [29] and the amplicons contained the NF-κB binding sites. A 227-bp fragment of the EP3 promoter was amplified by PCR with the primers sense 5′TTGTGGTTGAAGAAGCAGGAGGAC3′ and antisense 5′CTCTCTTGGCTGTTGCCTATCCTG 3′. A 158-bp fragment of the EP3 promoter was amplified by real-time PCR with the primers sense 5′ TCCACCGACACTTTAGCGGAGAAC3′ and antisense 5′ GAGGAGGGTGCAAAGCAACTGAGC3′.

### Statistical analyses

The data are shown as the mean ±SD and were analyzed using one-way ANOVA and independent sample *t*-test as appropriate. Each assay was performed at least 3 times and representative results are shown. P<0.05 was considered statistically significant.

## Results

### Immunolocalization of EP3 and NF-κB in human atherosclerotic plaques

**Figure 1:**
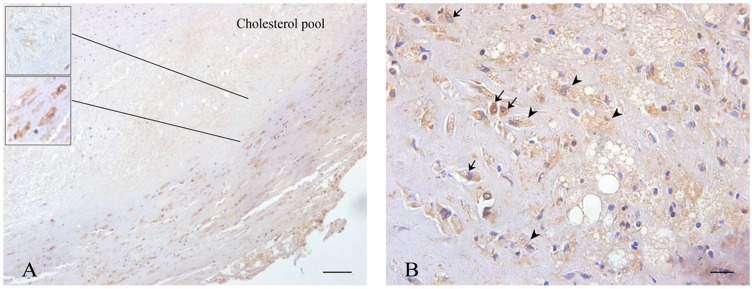
Immunohistochemical detection of EP3 expression in human atherosclerotic plaques. Arrows indicate macrophages; arrowheads indicate foam cells. Scale bar = 100 µm (A), 20 µm (B).

Previous studies have shown that EP3 is predominantly expressed by macrophages in the shoulder regions of atherosclerotic lesions [11]. Therefore, we first analyzed the expression and localization of EP3 by immunohistochemistry in 6 cases of human aortic atherosclerotic plaques. EP3 was mainly located in the inflammatory region, rather than the inner region of human atherosclerotic plaques (Fig. 1A). EP3, a seven-transmembrane receptor, was intensely expressed in the cytoplasm and was higher in macrophages than in foam cells (Fig. 1B).

### OxLDL suppresses EP3 expression in THP-1 macrophages

**Figure 2:**
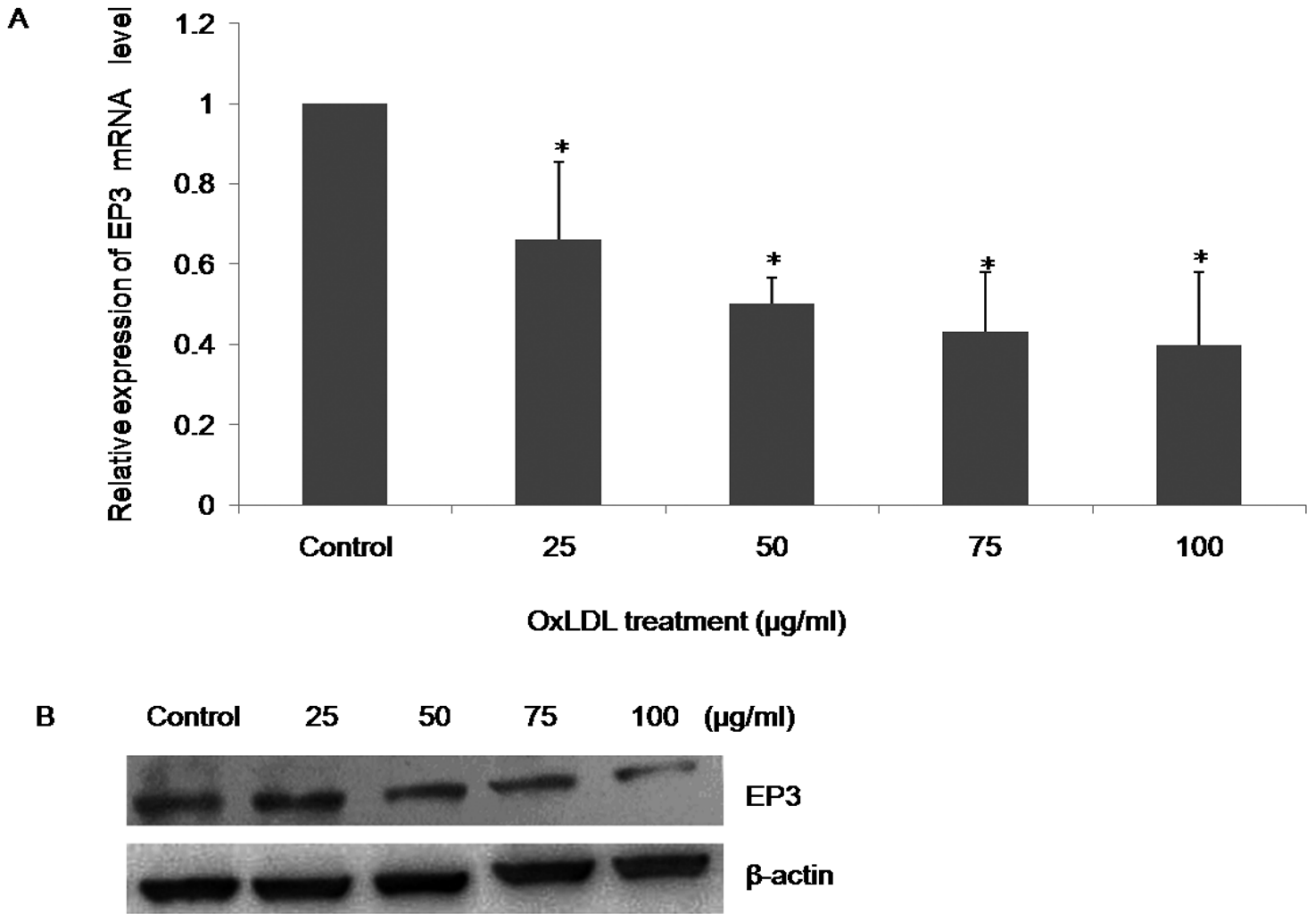
Effects of oxLDL on EP3 mRNA and protein expression in THP-1 macrophages. (A) Real-time PCR analysis of EP3 mRNA expression in THP-1 macrophages treated with oxLDL at various concentrations for 24 h. (B) Western blot analysis of EP3 protein expression in THP-1 macrophages exposed to oxLDL for 24 h. The representative image is shown. *P<0.05 versus control.

CD68 is a scavenger receptor expressed in the surface of mature macrophages [32]. PMA-differentiated THP-1 macrophages express CD68 protein (Fig. S1A). The effect of oxLDL on EP3 expression was evaluated in THP-1 macrophages by incubating cells with increasing concentrations of oxLDL for 24 h. The mRNA level of EP3 dose-dependently decreased with 25, 50, 75 and 100 µg/ml of oxLDL compared with the untreated control (Fig. 2A). Changes in protein expression revealed a similar pattern (Fig. 2B).

Oil-red O staining was used to detect intracytoplasmic lipid droplets. THP-1 macrophages treated with 50 µg/ml oxLDL for 24 h stained strongly with Oil-red O and quantification revealed greater staining in oxLDL-treated THP-1 macrophages than in untreated controls (Fig. S1B). Therefore, we used a 24-h exposure to 50 µg/ml for subsequent experiments.

### PPAR-γ regulates EP3 expression by oxLDL treatment in THP-1 macrophages

**Figure 3:**
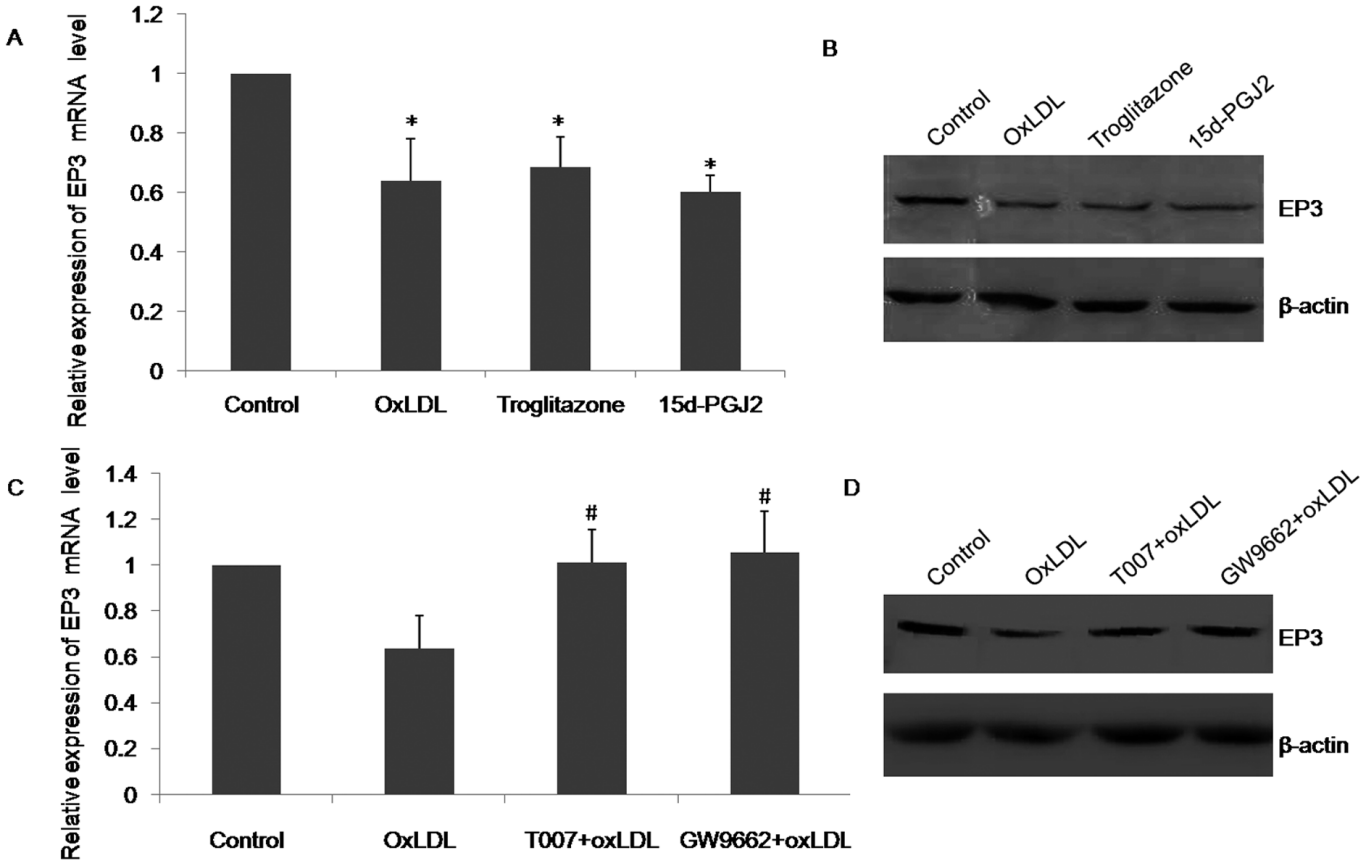
Effect of PPAR-γ agonists and antagonists on EP3 expression. (A) Real-time PCR analysis of EP3 mRNA expression with oxLDL (50 µg/ml), troglitazone (10 µM) or 15d-PGJ2 (1 µM) for 24 h. Data are from 3 experiments. * P<0.05 versus control. (B) Western blot analysis of EP3 protein levels following treatment with oxLDL (50 µg/ml), troglitazone (10 µM) or 15d-PGJ2 (1 µM) for 24 h. The representative image is shown. (C) Real-time PCR analysis of EP3 mRNA expression in THP-1 macrophages pretreated with T007 (5 µM) or GW9662 (5 µM) for 3 h, followed by addition of oxLDL (50 µg/ml) for 24 h. # p<0.05 versus oxLDL. (D) Western blot analysis of EP3 protein levels in THP-1 macrophages pretreated with T007 (5 µM) or GW9662 (5 µM) for 3 h, followed by addition of oxLDL (50 µg/ml) for 24 h. The representative image is shown.

Previous studies demonstrate that components of the oxLDL particle (9- or 13- hydroxyoctadecadienoic acid) serve as endogenous ligands of PPAR-γ to regulate macrophage gene expression [14]. We next determined whether PPAR-γ takes part in the regulation of oxLDL-inhibited EP3 expression, using PPAR-γ agonists, troglitazone and 15d-PGJ2. Consistent with oxLDL, both troglitazone and 15d-PGJ2 decreased EP3 mRNA and protein expression compared with the untreated control (Fig. 3A, B).

To further evaluate the inhibitory effect of PPAR-γ and oxLDL on EP3 expression, THP-1 macrophages were pretreated with PPAR-γ antagonists, T007 or GW9662, for 3 h prior to addition of oxLDL. Both T007 and GW9662 blocked oxLDL-mediated down-regulation of EP3 expression at both mRNA and protein levels (Fig. 3C, D). Thus, these results indicate that the inhibitory effect of oxLDL on EP3 expression is through up-regulating PPAR-γ.

### OxLDL decreases NF-κB activity in THP-1 macrophages

**Figure 4:**
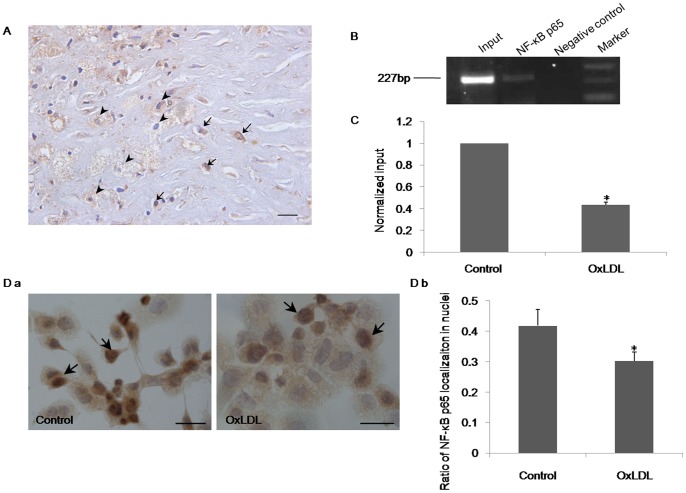
NF-κB regulates oxLDL-inhibited EP3 expression. (A) Immunohistochemical detection of NF-κB expression in human atherosclerotic plaques. Arrows indicate macrophages; arrowheads indicate foam cells. Scale bar = 20 µm. (B) PCR analysis of the binding of NF-κB to the EP3 promoter using a ChIP assay. The amplified EP3 promoter was 227 bp**.** (C) Real-time PCR analysis in a ChIP assay for the binding of NF-κB to the EP3 promoter in THP-1 macrophages treated with oxLDL (50 µg/ml) for 24 h. (D-a) Immunocytochemical analysis of NF-κB nuclear expression in THP-1 macrophages treated with oxLDL (50 µg/ml) for 24 h. Scale bar = 50 µm. (D-b) Statistical analysis of the percentage of nuclear-localized NF-κB in THP-1 macrophages treated with oxLDL (50 µg/ml) for 24 h. Data are from 3 separate experiments. * P<0.05 versus control.

The promoter sequence of the EP3 gene contains *cis*-acting elements for the transcription factor NF-κB [29]. Additionally, NF-κB activation (nuclear staining-positive) was higher in macrophages than in foam cells (Fig. 4A). The localization of NF-κB was similar to EP3. Thus, we investigated whether NF-κB took part in the regulation of oxLDL-inhibited EP3 expression in THP-1 macrophages. First, we used a ChIP assay to examine the binding activity of NF-κB subunit p65 to its recognition motif in THP-1 macrophages (Fig. 4B). Binding of NF-κB to the EP3 promoter was reduced in oxLDL-treated THP-1 macrophages compared to control (Fig. 4C). Furthermore, immunocytochemistry analysis showed that oxLDL treatment for 24 h decreased nuclear NF-κB expression in THP-1 macrophages compared with the untreated control (Fig. 4D). These results indicate that oxLDL decreases EP3 expression through inhibition of NF-κB nuclear translocation and binding to the EP3 promoter.

### PPAR-γ regulates EP3 expression via inhibition of NF-κB activity in THP-1 macrophages

**Figure 5:**
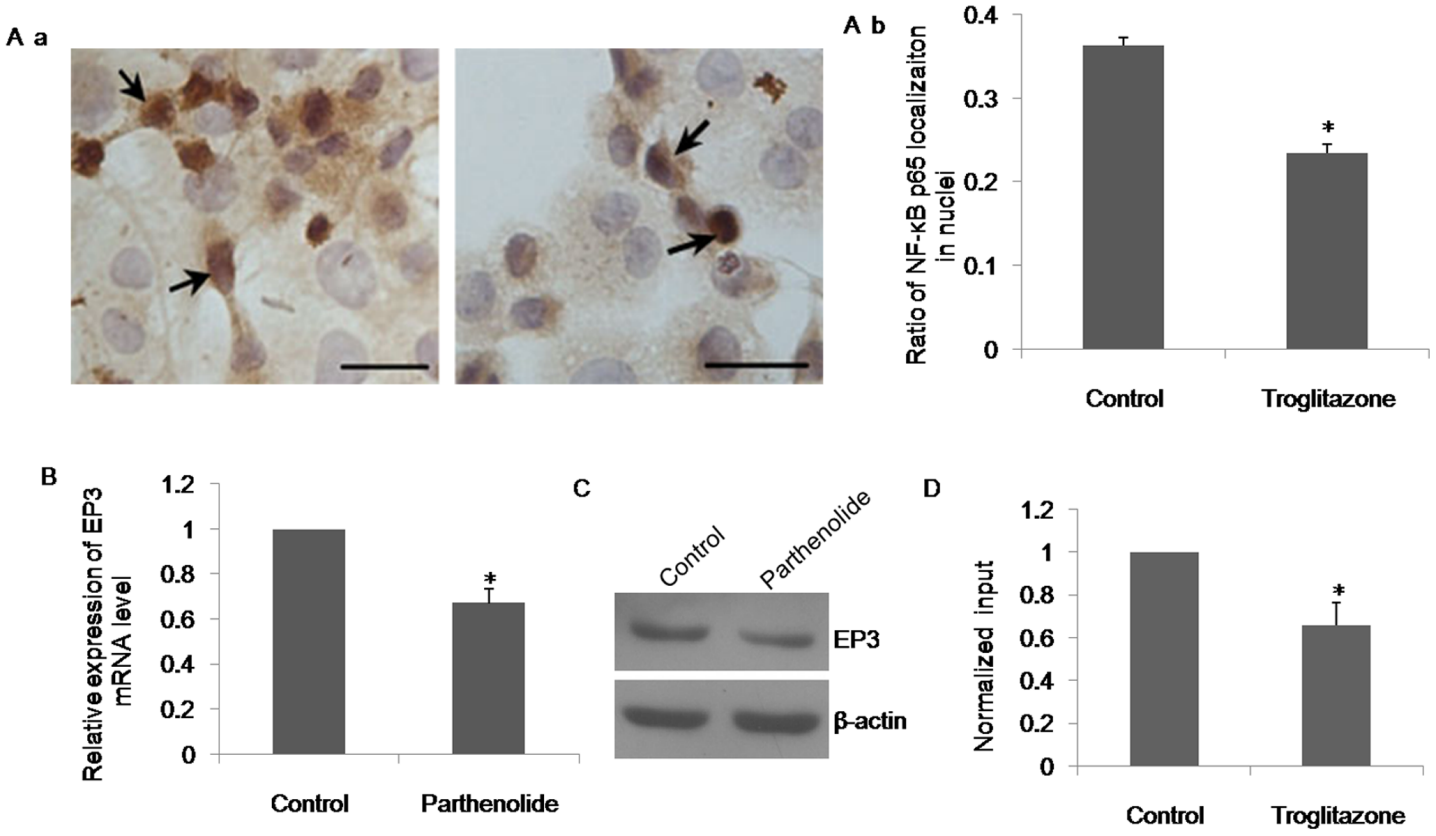
PPAR-γ activation inhibits EP3 expression by inhibiting NF-κB activity. (A-a) Immunocytochemical analysis of nuclear NF-κB expression in THP-1 macrophages treated with the PPAR-γ agonist, troglitazone (10 µM), for 24 h. Scale bar = 50 µm. (A-b) Statistical analysis of the number of nuclear NF-κB-positive cells based on the immunocytochemical results. (B) Real-time PCR analysis of EP3 mRNA expression following addition of parthenolide (5 µM) for 24 h. (C) Western blot analysis of EP3 protein expression with parthenolide (5 µM) for 24 h. (D) Real-time PCR analysis of the ChIP assay examining the binding of NF-κB to EP3 in THP-1 macrophages treated with troglitazone (10 µM) for 24 h. Data are from 3 experiments**.** * P<0.05 versus control.

To determine whether PPAR γ-reduced EP3 expression was regulated by NF-κB, we examined NF-κB nuclear expression and its binding activity to the EP3 promoter in THP-1 macrophages treated with PPAR-γ agonists. Addition of troglitazone decreased nuclear NF-κB expression (Fig. 5A). Parthenolide, an NF-κB inhibitor, also decreased EP3 mRNA and protein expression (Fig. 5B, C). Additionally, a ChIP assay revealed that the binding of NF-κB to the EP3 promoter was decreased in THP-1 macrophages incubated with troglitazone relative to the control (Fig. 5D). These results indicate that PPAR-γ agonists decrease EP3 expression by inhibiting NF-κB nuclear translocation and the binding of NF-κB to the EP3 promoter.

## Discussion

Atherosclerosis is a chronic inflammatory disease as well as a metabolic lipid disorder [33]. Atherosclerotic lesions are characterized by the infiltration of macrophages that internalize oxLDL to form foam cells [34]. Although Cipollone and co-workers do not detect EP3 expression in atherosclerotic plaques [10], others have demonstrated [11], [12], and we demonstrate here that EP3 expression is mainly located in the inflammatory region and is higher in macrophages than in foam cells of human atherosclerotic plaques (Fig. 1). However, the mechanism behind this EP3 expression pattern has not yet been elucidated.

We find that oxLDL suppresses EP3 expression in THP-1 macrophages (Fig. 2). Prior investigators have shown that components of the oxLDL particle (9- or 13-hydroxyoctadecadienoic acid) are endogenous ligands of PPAR-γ and oxLDL regulates macrophage gene expression through activation of PPAR-γ [14]. PPAR-γ was expressed in macrophages of atherosclerotic lesion [35], but whether PPAR-γ was involved in the regulation of EP3 expression was unknown. We find that PPAR-γ agonists, troglitazone and 15d-PGJ2, inhibits EP3 expression like oxLDL and PPAR-γ antagonists block oxLDL-mediated down-regulation of EP3 (Fig. 3). These data indicate that oxLDL suppresses EP3 expression through activation of PPAR-γ. The role of PPAR-γ in modulating the development and progression of atherosclerosis is contradictory and activation of PPAR-γ receptors has both favorable and unfavorable effects on cardiovascular processes [36]. Our results, that PPAR-γ agonists inhibit EP3 expression, reflect the unfavorable effects of PPAR-γ in atherosclerosis.

Previous studies have shown that oxLDL may activate or inhibit NF-κB activation depending on whether or not the cells are activated, extent of LDL oxidation and incubation time. Short-term (4 hours) incubations or use of low oxLDL concentrations activate NF-κB in monocytes [37]. However, long-term (>20 hours) incubations or use of more severely oxidized LDL inhibit NF-κB activity in monocytes and macrophages [37], [38]. OxLDL activates NF-κB to induce gene expression in resting macrophages [39], whereas it inhibits NF-κB to suppress gene expression in LPS-activated macrophages [21], [40], [41]. PMA also bears some common features with LPS and can activate macrophages [42], [43]. We find oxLDL decreases NF-κB nuclear translocation and binding of NF-κB to the EP3 promoter in PMA-differentiated THP-1 macrophages (Fig. 4B–D). This suggests that oxLDL suppresses EP3 expression by inhibiting NF-κB activity.

Several studies have shown that PPAR-γ inhibits gene expression though antagonizing the activity of NF-κB [20], [44], [45]. The EP3 promoter contains *cis*-acting elements for the transcription factor NF-κB but not for PPAR-γ [29]. OxLDL suppressed the IL-12 production by inhibiting the NF-κB-DNA interactions and interactions between NF-κB and PPAR-γ in activated macrophages [21]. We therefore investigated whether the same finding could be observed in oxLDL-inhibited EP3 expression. We find PPAR-γ agonists decrease NF-κB nuclear translocation and the binding of NF-κB to the EP3 promoter (Fig. 5). These results indicate that oxLDL suppresses EP3 expression by PPAR γ-mediated inhibition of NF-κB activity.

It was reported that EP3 mRNA levels were significantly lower in plaques than in their adjacent regions or in control arteries [13]. The PGE_2_-EP3 pathway had an anti-inflammatory effect in allergic inflammation [46], [47]. Our results that oxLDL inhibits EP3 expression suggest that the EP3 receptor may exert a beneficial effect on atherosclerosis.

In summary, we find that oxLDL inhibits EP3 expression in THP-1 macrophages, which explains why EP3 expression is higher in THP-1 macrophages than in foam cells. We further demonstrate that oxLDL suppresses EP3 expression by activation of PPAR-γ and subsequent inhibition of NF-κB activity in THP-1 macrophages.

## Supporting Information

Figure S1 **Detection of THP-1 macrophages and foam cells.** (A) Immunohistochemical detection of CD68 expression in THP-1 macrophages treated with PMA for 24 h. Original magnification ×400. (B) Oil-red O staining in THP-1 macrophages with or without 50 µg/ml of oxLDL treatment for 24 h. Representative images are shown. Original magnification ×200.
(TIF)
