# Supplementary figures and images for: Oxidized Low-Density Lipoprotein Suppresses Expression of Prostaglandin E Receptor Subtype EP3 in Human THP-1 Macrophages
Source: PLoS One. 2014 Oct 21;9(10):e110828. doi: 10.1371/journal.pone.0110828 (PMC4205008; doi:10.1371/journal.pone.0110828)

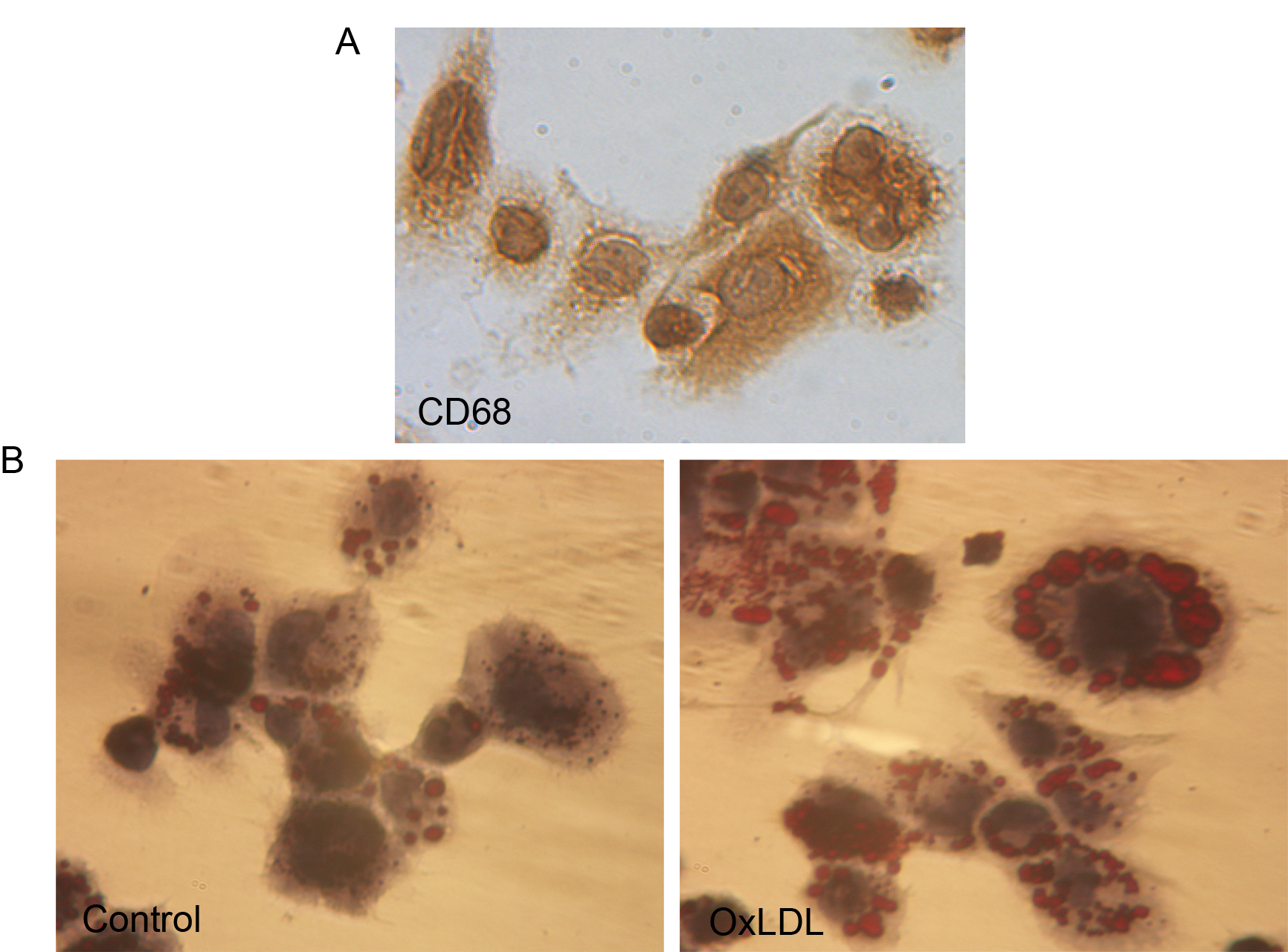

Supplement: Figure S1 — Detection of THP-1 macrophages and foam cells. (A) Immunohistochemical detection of CD68 expression in THP-1 macrophages treated with PMA for 24 h. Original magnification ×400. (B) Oil-red O staining in THP-1 macrophages with or without 50 µg/ml of oxLDL treatment for 24 h. Representative images are shown. Original magnification ×200. (TIF) [file pone.0110828.s001.tif]
